# Comparing Implant Stability Following Conventional Osteotomy and Osseodensification in Contrasting Ridge Conditions: Report of a Case Treated Using the Split-Mouth Technique

**DOI:** 10.7759/cureus.112538

**Published:** 2026-07-12

**Authors:** D. Harishkumar Alias Nagarajan, Jasmin Raichel Kunnumpuram, Kennedy Babu, Soorya K.V., Manoj Margabandhu, Lakshminarayanan S

**Affiliations:** 1 Periodontology, Mahatma Gandhi Postgraduate Institute of Dental Sciences, Puducherry, IND

**Keywords:** implant stability, implant stability quotient (isq), implant success, ridge preservation, surgical case reports

## Abstract

Primary implant stability is central to predictable osseointegration and long-term implant success. While conventional subtractive osteotomy remains a reliable approach in ridges of adequate width, osseodensification has been proposed as a bone-preserving alternative, particularly in narrower ridges. However, direct intra-patient comparisons under contrasting ridge conditions remain relatively limited. This report describes a 35-year-old systemically healthy female presenting with bilateral mandibular first molar edentulism. Preoperative cone-beam computed tomography demonstrated a ridge width of 7 mm with D3 bone density at site 46 and 5 mm with D2 bone density at site 36. A flapless conventional osteotomy was performed at site 46, whereas site 36 underwent flap elevation followed by osseodensification-assisted preparation to allow controlled ridge expansion. Implant stability was assessed using resonance frequency analysis.

Primary stability measured 72 implant stability quotient (ISQ) at site 46 and 75 ISQ at site 36. At 16 weeks, both implants demonstrated secondary stability values of 71 ISQ. Radiographic evaluation at prosthetic loading revealed minimal crestal bone remodeling (≤0.5 mm at all measured surfaces), with no evidence of peri-implant radiolucency. Healing progressed without complication. Within the inherent limitations of a single-patient case report, osseodensification in a narrow ridge achieved stability comparable to flapless conventional osteotomy performed in an anatomically favorable ridge.

## Introduction

Primary implant stability plays a decisive role in successful osseointegration and long-term implant survival [1]. Stability is influenced by bone density, ridge morphology, implant macrodesign, and surgical technique. Conventional subtractive osteotomy removes bone to create implant space and is predictable in ridges with sufficient width. However, this technique may reduce peri-implant bone mass. Osseodensification is a non-subtractive drilling method that compacts trabecular bone laterally, thereby increasing peri-implant bone density and mechanical engagement [2,3]. Histologic studies have demonstrated improved bone-to-implant contact and enhanced insertion torque when densification techniques are employed [3,4].

Clinical investigations have shown that osseodensification can facilitate ridge expansion through controlled plastic deformation, particularly in narrow alveolar ridges [5]. Randomized clinical trials comparing osseodensification with conventional drilling in compromised ridges have reported favorable early stability outcomes with densification protocols [6]. Flapless implant surgery, when ridge morphology permits, has been associated with preservation of periosteal blood supply and reduced surgical morbidity, potentially supporting favorable healing responses [7,8].

Although conventional osteotomy and osseodensification are individually well documented in the literature, direct intra-patient comparisons remain limited, particularly in situations where the osteotomy approach is selected according to deliberately contrasting ridge morphology and bone quality. Importantly, the two implant sites described in this split-mouth case were not anatomically identical at baseline. Site 46 exhibited a comparatively favorable ridge configuration with a width of 7 mm and bone quality consistent with D3, whereas site 36 presented with a narrower ridge width of 5 mm and relatively denser D2 bone. These site-specific differences were recognized preoperatively and directly influenced the surgical approach selected for each site, with flapless conventional osteotomy performed in the wider ridge and osseodensification-assisted preparation with ridge expansion undertaken in the narrower ridge. Accordingly, the present report is intended to illustrate the clinical behavior of two osteotomy strategies under intentionally contrasting anatomical conditions within the same patient, rather than to present a controlled comparison between two equivalent implant sites.

Primary implant stability represents the immediate mechanical engagement between the implant surface and the surrounding bone at the time of placement. It is widely considered a fundamental prerequisite for predictable osseointegration [1]. Several factors contribute to this initial stability, including bone density, ridge morphology, implant design characteristics, and the surgical technique employed.

Conventional subtractive osteotomy creates the implant bed through sequential bone removal. Although dependable in ridges of sufficient width, this approach may reduce peri-implant bone volume. In contrast, osseodensification utilizes specially designed burs operated in a non-cutting mode to compact trabecular bone laterally rather than remove it. This concept has been associated with increased peri-implant bone density and improved mechanical engagement [2,3], and histologic investigations have demonstrated enhanced bone-to-implant contact with densification protocols [3,4].

Clinical reports suggest that osseodensification may permit controlled ridge expansion through plastic deformation of trabecular bone, particularly in narrow alveolar ridges [5]. Randomized trials have also demonstrated improved early stability values when densification is applied in compromised ridge conditions [6]. When ridge dimensions permit, flapless implant placement has been associated with preservation of periosteal blood supply and reduced surgical morbidity [7,8]. Although each of these techniques is individually supported in the literature, direct intra-patient comparisons under differing ridge morphologies remain limited. This split-mouth case was undertaken to evaluate implant stability outcomes under such contrasting anatomical conditions within the same patient.

## Case presentation

A 35-year-old systemically healthy female presented with missing mandibular first molars (36 and 46), previously extracted due to caries. Clinical examination revealed adequate keratinized tissue and the absence of active periodontal disease (Figure 1). No parafunctional habits were reported. Given the contrasting ridge anatomy, site 46 was planned for flapless conventional osteotomy, while site 36 was planned for flap-elevated osseodensification-assisted osteotomy with controlled ridge expansion. Preoperative CBCT was used to assess ridge width and bone quality at both sites. Bone quality was categorized according to the Lekholm and Zarb classification, based on the radiographic trabecular pattern and cortical bone characteristics observed on CBCT.

**Figure 1:**
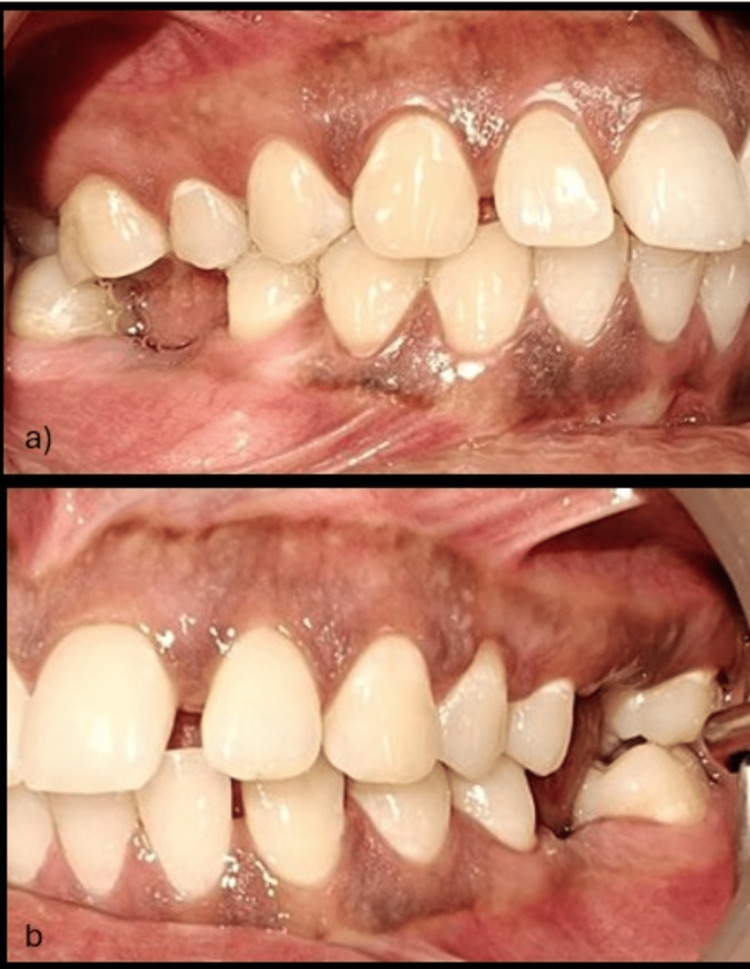
Preoperative lateral views of the patient's mouth The image shows missing mandibular first molars (a) right lateral view (b) left lateral view.

Site 46 (Figure 2) demonstrated a ridge width of 7 mm with D3 bone quality, whereas site 36 demonstrated a ridge width of 5 mm with D2 bone quality (Figure 3). The case is reported in compliance with CARE guidelines.

**Figure 2:**
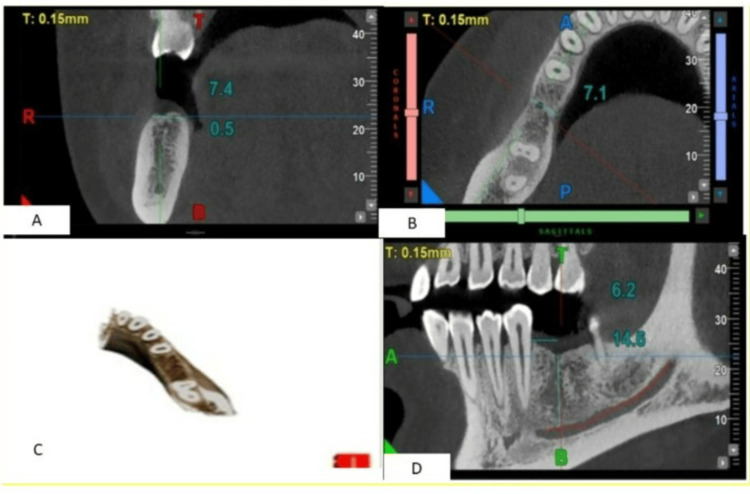
CBCT evaluation images of site 46 Cone-beam computed tomography (CBCT) evaluation of site 46 (A) cross-sectional (coronal) view, (B) axial view, (C) 3D volumetric reconstruction, and (D) sagittal (mesiodistal) view.

**Figure 3:**
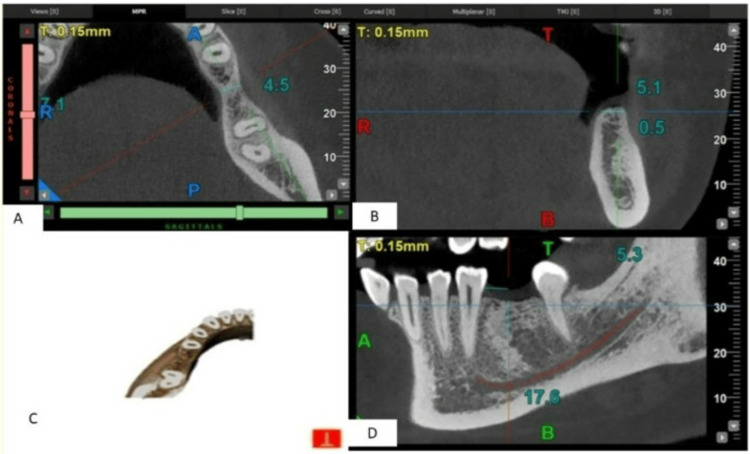
CBCT evaluation images of site 36 Cone-beam computed tomography evaluation of site 36. (A) axial view, (B) cross-sectional (coronal) view, (C) 3D volumetric reconstruction, and (D) sagittal (mesiodistal) view.

Surgical procedure

All procedures were performed under local anesthesia using 2% lidocaine with 1:100,000 epinephrine. Implant placement at both sites was carried out using a freehand approach, guided by preoperative CBCT evaluation and intraoperative clinical judgment. A static surgical guide was not utilized because the present case was intended to reflect routine clinical implant placement under two contrasting ridge conditions rather than a fully standardized guided protocol. In addition, direct intraoperative assessment of crestal anatomy and osteotomy orientation was considered particularly important at site 36, where flap elevation and osseodensification-assisted ridge expansion were performed in a narrow ridge. Osteotomies were prepared at 800-1500 rpm under continuous saline irrigation to minimize thermal trauma.

Site 46 - Flapless Conventional Osteotomy

A tissue punch was used to access the crestal bone without raising a flap, allowing preservation of the periosteal blood supply. Sequential osteotomy preparation was completed according to the manufacturer’s protocol. A 4.0 × 11.5 mm implant was inserted and achieved a peak insertion torque of 40 N·cm. This torque value reflected satisfactory mechanical engagement within the D3 bone at this site and was considered appropriate for achieving reliable primary stability in a ridge of adequate width. Resonance frequency analysis performed immediately after placement demonstrated a primary stability value of 72 ISQ (Figures 4A-4C), corroborating the clinical perception of stable implant fixation.

**Figure 4:**
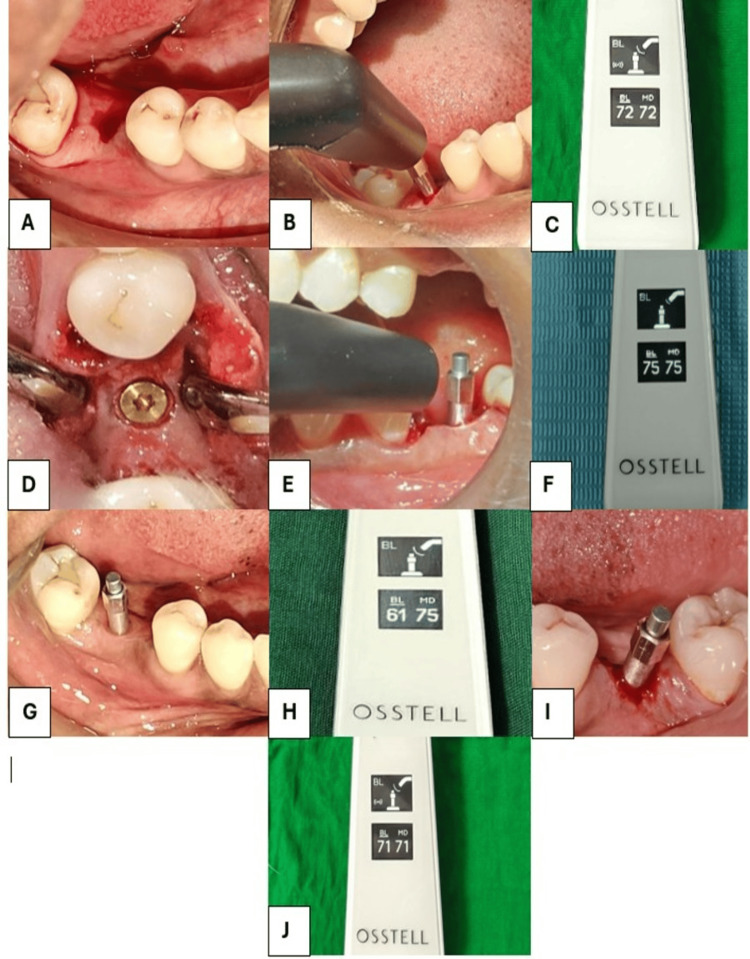
Treatment procedure images (A) Flapless conventional osteotomy at site 46, (B, C) primary stability evaluation of 46, (D) flap-elevated osseodensification at site 36, (E, F) Primary stability evaluation of 36, (G, H) secondary stability evaluation of 46, (I, J) secondary stability evaluation of 36.

Site 36 - Flap-Elevated Osseodensification

Given the reduced ridge width, a full-thickness mucoperiosteal flap was carefully elevated to allow direct visualization of the crestal anatomy. Osteotomy preparation was performed using the Densah™ bur system (Versah LLC, USA) in accordance with established osseodensification principles [2]. A pilot drill was initially used in clockwise (cutting) mode to establish the osteotomy trajectory. Subsequent osteotomy enlargement was carried out using Densah burs (2.0 mm, 2.3 mm, and 3.0 mm) operated in counter-clockwise (non-cutting densifying mode) at 800-1200 rpm with copious irrigation. This approach allowed controlled lateral bone compaction rather than bone removal. The final osteotomy diameter was approximately 3.0 mm, intentionally underprepared relative to the 3.5 mm implant diameter to enhance mechanical engagement within the available bone. Clinically, controlled expansion of the crestal ridge was observed, increasing from approximately 5 mm preoperatively to nearly 7 mm found from the CBCT radiographs following densification, consistent with previously reported observations [5].

A 3.5 × 11.5 mm implant was then inserted, achieving a peak insertion torque of 45 N cm, reflecting firm mechanical engagement within the densified bone. Resonance frequency analysis performed immediately after placement demonstrated a primary stability value of 75 ISQ (Figures 4D-4F). Following flap reflection, the pre-expansion buccolingual ridge width at site 36 was assessed clinically at the crestal level using a UNC-15 periodontal probe and a bone gauge/caliper. After completion of osseodensification-assisted osteotomy preparation and controlled ridge expansion, the ridge width was reassessed at the same crestal reference level using the same instruments to maintain consistency of measurement. The increase in ridge width was therefore based on direct intraoperative clinical measurement rather than radiographic estimation alone. Because flap elevation and osseodensification-assisted ridge expansion had been performed in a narrow ridge, the implant at site 36 was initially submerged with a cover screw to permit undisturbed healing and tension-free flap closure over the expanded ridge. A healing abutment was placed at a later stage during the healing phase prior to prosthetic progression.

Postoperative care and evaluation

Amoxicillin 500 mg with clavulanic acid 125 mg was administered every 12 hours for five days, along with paracetamol 650 mg as required for pain control. 0.2% chlorhexidine mouth rinse twice daily for two weeks was also prescribed. Primary implant stability was assessed immediately after placement using resonance frequency analysis (RFA). The implant placed at site 46 through a flapless conventional osteotomy demonstrated a primary stability value of 72 ISQ. At site 36, following flap elevation and osseodensification, primary stability measured 73 ISQ. Clinically, ridge width at site 36 increased from approximately 5 mm preoperatively to nearly 7 mm after controlled densification.

Healing was uneventful at both sites. The patient reported no unusual discomfort, and clinical follow-up revealed healthy peri-implant soft tissues without signs of inflammation, dehiscence, or infection. Secondary stability was assessed at 16 weeks using resonance frequency analysis, with both implants demonstrating stable readings of 71 ISQ (Figures 4G-4J). Standardized periapical radiographic evaluation at the time of prosthetic loading revealed minimal crestal bone remodeling. At site 46, the crestal bone level measured approximately 0.4 mm mesially and 0.5 mm distally from the implant platform. At site 36, corresponding measurements were 0.3 mm mesially and 0.4 mm distally. These findings indicated the preservation of marginal bone levels, with no evidence of peri-implant radiolucency.

Three months follow-up

Three-month postoperative radiographic evaluation at site 46 demonstrated maintained crestal bone levels (Figure 5). Three-month postoperative radiographic evaluation at site 36 similarly demonstrated preserved marginal bone levels (Figure 6). Both implants demonstrated stable functional performance and well-maintained peri-implant soft tissue contours.

**Figure 5:**
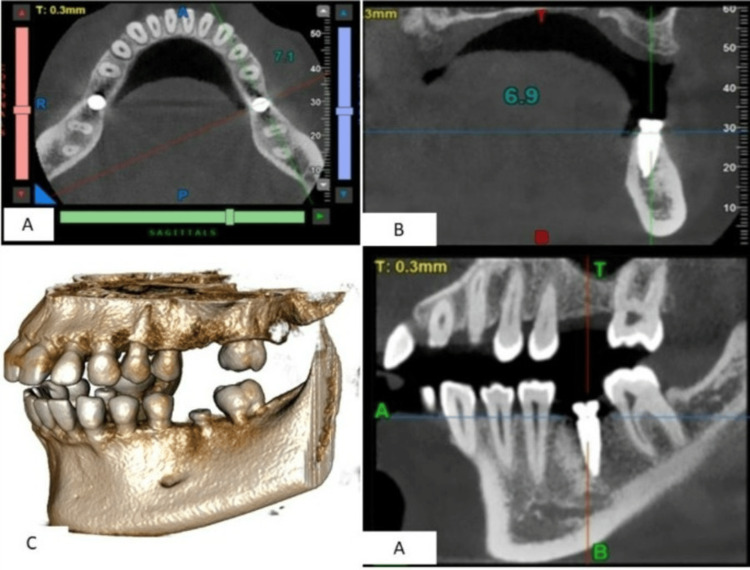
Three-month postoperative radiographic follow-up at site 46 (A) Axial view, (B) cross-sectional (coronal) view, (C) 3D volumetric reconstruction (D) sagittal (mesiodistal) view.

**Figure 6:**
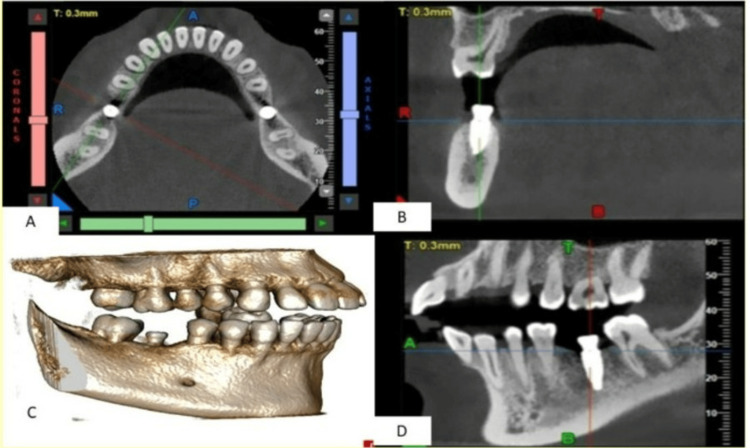
Three-month postoperative radiographic follow-up at site 36 (A) Axial view, (B) cross-sectional (coronal) view, (C) 3D volumetric reconstruction, (D) sagittal (mesiodistal) view.

A comprehensive table describing the clinical and surgical timeline is presented in Table 1.

**Table 1:** Clinical and surgical timeline for the patient

Time point	Clinical event/assessment
Initial presentation	A 35-year-old systemically healthy female presented with bilateral mandibular first molar edentulism at sites 36 and 46 following extraction due to caries. Clinical examination revealed adequate keratinized tissue and absence of active periodontal disease.
Preoperative assessment	Preoperative CBCT evaluation was performed to assess ridge dimensions, bone architecture, and implant planning at both sites. Site 46 demonstrated a ridge width of approximately 7 mm with bone quality consistent with D3, whereas site 36 demonstrated a ridge width of approximately 5 mm with bone quality consistent with D2.
Surgical planning	Based on the contrasting ridge anatomy, site 46 was planned for flapless conventional osteotomy, while site 36 was planned for flap-elevated osseodensification-assisted osteotomy with controlled ridge expansion.
Surgical phase – Site 46	A flapless conventional osteotomy was performed at site 46 using a tissue punch and sequential osteotomy preparation according to the manufacturer’s drilling protocol. A 4.0 × 11.5 mm implant was placed, achieving a peak insertion torque of 40 Ncm. Primary implant stability measured by resonance frequency analysis (RFA) was 72 ISQ. A healing abutment was placed immediately to allow transmucosal healing.
Surgical phase – Site 36	A full-thickness mucoperiosteal flap was elevated at site 36. The pre-expansion ridge width was assessed clinically at the crestal level using a UNC-15 periodontal probe and bone gauge/caliper. Osseodensification-assisted osteotomy was then performed using Densah™ burs in densifying mode, resulting in controlled ridge expansion from approximately 5 mm to nearly 7 mm. A 3.5 × 11.5 mm implant was placed with a peak insertion torque of 45 N.cm. Primary stability as measured by RFA was 75 ISQ. The implant was initially submerged with a cover screw to permit undisturbed healing and flap closure.
Healing phase	Site 46 continued healing transmucosally with the healing abutment in place. At site 36, the submerged implant was allowed to heal beneath the flap, and the cover screw was later replaced with a healing abutment during the healing phase prior to prosthetic progression.
16-week/three-month follow-up	Secondary implant stability was assessed at 16 weeks using RFA. Both implants demonstrated stable secondary stability values of 71 ISQ. Postoperative CBCT evaluation was used to assess implant positioning and peri-implant bone response, which demonstrated satisfactory implant positioning, maintained peri-implant bone levels, and absence of peri-implant radiolucency.

## Discussion

Primary implant stability is inherently multifactorial, reflecting interactions among bone quality, ridge dimensions, insertion torque, and surgical preparation technique [1]. Osseodensification alters the conventional osteotomy concept by compacting rather than removing bone, thereby potentially enhancing peri-implant bone density and mechanical interlocking [2,3]. Kassem et al. also reported higher primary stability with osseodensification compared to the conventional drilling method [4]. However, considering secondary stability, Kassem et al. assessed it after six months, whereas in this case, stability was obtained within 16 weeks [4]. Regarding mechanical engagement, Bergamo et al. conducted a split-mouth maxillary study and found that the ISQ value and insertion torque improved significantly with osseodensification compared with the conventional method. In the present case, despite the narrower ridge at site 36, the primary and secondary stability values were comparable to those achieved at site 46, where ridge dimensions were more favorable. In this study, the ISQ value decreased after 16 weeks for drilling, whereas for Bergamo et al., it remained unchanged [5].

The clinically observed increase in ridge width from approximately 5 mm to nearly 7 mm following densification aligns with previously reported observations of controlled plastic deformation [6]. Interestingly, insertion torque was slightly higher in the densified site (45 Ncm vs. 40 Ncm), suggesting enhanced mechanical engagement. The ridge expansion of 2 mm found in this study was consistent with the ridge expansion approximately reported by Guner and Canakci [7]. Although the stability findings contradicted those of Guner and Canakci, Hammouda et al. similarly found appropriate ridge expansion, but the primary stability was comparable between osseodensification as well as in the drilling method [8].

In this case, site 36 exhibited D2 bone density compared with D3 at site 46, which may have contributed to the observed stability values. This underscores the complexity of isolating the effect of the surgical technique alone. However, a low-density model study conducted by Arpudaswamy et al. found no difference in primary stability despite the improvement reported in mechanical engagement, such as the insertion torque [9]. Importantly, early marginal bone remodeling remained minimal at both sites, suggesting that neither technique adversely influenced crestal bone levels in the short term. The long-term remodeling favored healing and mechanical engagement [10-12]. This study included both anatomical conditions, such as a favorable ridge and a non-favorable ridge, together, and so it was preferable to study osseodensification on the non-favorable ridge and the conventional method on the favorable ridge. Since there was only one case, multiple factors, such as oral hygiene and healing, were controlled and comparable.

The present report has several limitations that should be considered when interpreting the findings. First, this was a single-patient split-mouth case report; therefore, no statistical analysis could be performed. The findings should accordingly be interpreted as descriptive clinical observations rather than statistically validated comparative outcomes. Second, the two implant sites were intentionally different in ridge width and bone quality, with site 46 presenting a wider ridge with D3 bone quality and site 36 a narrower ridge with D2 bone quality. These baseline anatomical differences may themselves have influenced implant stability outcomes independent of the osteotomy technique. Third, the implants placed at the two sites were not identical in diameter: a 4.0 × 11.5 mm implant at site 46 and a 3.5 × 11.5 mm implant at site 36; this may have affected the degree of bone-implant contact, insertion torque, and resonance frequency analysis values. In addition, different flap approaches were employed at the two sites: a flapless conventional osteotomy at site 46 and flap elevation with osseodensification-assisted ridge expansion at site 36. These procedural differences, together with the freehand placement approach and the differing healing protocols between the two sites, may have influenced implant angulation, peri-implant healing, and stability measurements. Furthermore, radiographic follow-up was performed using CBCT rather than serial standardized periapical radiographs, and the follow-up period was limited to 16 weeks, which restricts evaluation of longer-term crestal bone remodeling and implant behavior under functional loading and occlusion.

## Conclusions

The report of this split-mouth case demonstrates that osseodensification significantly improves mechanical engagement and primary stability in compromised anatomical conditions. After 16 weeks of healing, secondary stability was equalized between the two methods, demonstrating that the compromised site ultimately matched the stability of the favorable site. Ultimately, the choice of osteotomy technique should be individualized based on the patient's specific ridge morphology. While the baseline difference in bone density likely influenced the initial stability readings, the overall findings confirm that osseodensification can successfully compensate for unfavorable conditions, yielding predictable and successful outcomes comparable to conventional drilling in ideal bone.
